# Co-infection with iflaviruses influences the insecticidal properties of Spodoptera exigua multiple nucleopolyhedrovirus occlusion bodies: Implications for the production and biosecurity of baculovirus insecticides

**DOI:** 10.1371/journal.pone.0177301

**Published:** 2017-05-05

**Authors:** Arkaitz Carballo, Rosa Murillo, Agata Jakubowska, Salvador Herrero, Trevor Williams, Primitivo Caballero

**Affiliations:** 1 Instituto de Agrobiotecnología, CSIC-Gobierno de Navarra, Navarra, Spain; 2 Departamento de Producción Agraria, Universidad Pública de Navarra, Navarra, Spain; 3 Departamento de Genética, Universitat de Valencia, Valencia, Spain; 4 Instituto de Ecología AC, Xalapa, Mexico; Ecole des Mines d'Ales, FRANCE

## Abstract

Biological insecticides based on Spodoptera exigua multiple nucleopolyhedrovirus (SeMNPV) can efficiently control *S*. *exigua* larvae on field and greenhouse crops in many parts of the world. Spanish wild populations and laboratory colonies of *S*. *exigua* are infected by two iflaviruses (SeIV-1 and SeIV-2). Here we evaluated the effect of iflavirus co-infection on the insecticidal characteristics of SeMNPV occlusion bodies (OBs). Overall, iflavirus co-inoculation consistently reduced median lethal concentrations (LC_50_) for SeMNPV OBs compared to larvae infected with SeMNPV alone. However, the speed of kill of SeMNPV was similar in the presence or absence of the iflaviruses. A reduction of the weight gain (27%) associated with iflavirus infection resulted in a 30% reduction in total OB production per larva. Adult survivors of SeMNPV OB inoculation were examined for covert infection. SeMNPV DNA was found to be present at a high prevalence in all SeIV-1 and SeIV-2 co-infection treatments. Interestingly, co-inoculation of SeMNPV with SeIV-2 alone or in mixtures with SeIV-1 resulted in a significant increase in the SeMNPV load of sublethally infected adults, suggesting a role for SeIV-2 in vertical transmission or reactivation of sublethal SeMNPV infections. In conclusion, iflaviruses are not desirable in insect colonies used for large scale baculovirus production, as they may result in diminished larval growth, reduced OB production and, depending on their host-range, potential risks to non-target Lepidoptera.

## Introduction

Recent advances in next generation sequencing have led to the discovery of a great diversity of viruses infecting insect populations [1, 2]. RNA viruses belonging to the family *Iflaviridae* (order *Picornavirales*) were detected during analysis of the larval transcriptome [3] of the beet armyworm, *Spodoptera exigua* (Lepidoptera: Noctuidae), which is a major pest of horticultural crops in tropical and subtropical and regions worldwide [4]. This pest also attacks greenhouse grown vegetable and ornamental crops in temperate regions [5–7].

Iflaviruses exclusively infect arthropods [8] and although some can cause clear signs of disease in their hosts, such as the iflaviruses that infect *Bombyx mori* [9] and *Apis mellifera* [10], generally iflavirus infections remain unnoticed until detected using molecular tools [11]. Specifically, two iflaviruses were found to be prevalent in *S*. *exigua* natural populations in Spain, which were named Spodoptera exigua iflavirus-1 (SeIV-1) [12] and Spodoptera exigua iflavirus-2 (SeIV-2) [13]. SeIV-1 has a 10.3 kb genome whereas SeIV-2 has a 9.4 kb genome of ssRNA. In both cases the genomes are packaged into icosahedral capsids of 27 nm diameter. The genome consists of a single ORF which is translated as a single polyprotein that is subsequently cleaved into functional and structural proteins.

Baculoviruses (*Baculoviridae*) are rod-shaped DNA viruses that are occluded in proteinaceous occlusion bodies (OBs) that have been developed as the basis for effective insecticidal products in many countries [14]. The Spodoptera exigua multiple nucleopolyhedrovirus (SeMNPV) has been developed for the biological control of the beet armyworm in developed and developing countries due to its high specificity, pathogenicity and virulence [15]. In contrast, the use of RNA viruses as insect control agents has been limited to experimental studies [16], mainly due to their low virulence and also because of biosecurity concerns given their phylogenetic proximity to related viruses of the order *Picornavirales* that are human and animal pathogens [17].

Mixed infections comprising iflavirus and other viruses have been reported in insects [18, 11], in conjunction with baculoviruses [19–21] and a densovirus [22]. Field-caught adults of *S*. *exigua* were found to be infected with both SeMNPV and two iflaviruses, and their laboratory-reared offspring also showed a high prevalence of both viruses [23, 24]. Recent studies on the association between SeMNPV and iflaviruses revealed that genomes of both types of viruses were present in the OBs of SeMNPV produced in insects that were infected by both viruses. The OB structure improved the resistance of the iflavirus to UV light and high temperatures whereas a decrease in OB pathogenicity was observed in OBs produced in co-infected insects [25].

The key variables that influence the transmission of baculoviruses include the pathogenicity of OBs, measured in terms of concentration-mortality metrics, the speed of kill and the production of progeny OBs. These characteristics also determine the effectiveness of these viruses as biological insecticides [26]. As cell culture production results in rapid loss of auxiliary genes and reduced pathogenicity in insects, all baculovirus based insecticides are currently produced in lepidopteran larvae [27]. Efficient baculovirus production therefore relies on a large, productive and healthy insect colony. Viruses can cause devastating epizootics of disease in insect colonies and require continuous monitoring and disinfection processes to maintain colony health [28–30]. Iflaviruses are rapidly transmitted and can reach a high prevalence of infection in natural populations and laboratory insect colonies [11, 31, 32].

As iflaviruses are present in *S*. *exigua* populations used to produce SeMNPV as the basis for biological insecticides, we examined the consequences of coinfection with SeMNPV and SeIV-1 and SeIV-2 on the insecticidal properties of OBs produced in co-infected insects. The findings have clear relevance to the efficacy of baculovirus-based insecticides and the biosecurity and potential non-target effects of these products.

## Materials and methods

### Insects and virus stock

*Spodoptera exigua* virus-free insects used in the experiments were obtained from Entomotech (Almería, Spain) and Andermatt Biocontrol (Switzerland) as egg masses. Insects were reared on semi-artificial diet [33] at 25 ± 2°C, 50 ± 10% relative humidity and 16 h:8 h light:dark photoperiod in disinfected bioclimatic chambers used exclusively for this propose.

OBs of the Spanish isolate SeMNPV-SP2 were obtained from the baculovirus collection held at the Universidad Pública de Navarra since 1996 [34]. These OBs consistently proved negative for the presence of SeIV-1 and SeIV-2 by RT-qPCR [25]. SeIV-1 and SeIV-2 particles were isolated from larvae previously found to harbor a persistent infection. For this, virus particles were purified from guts dissected from groups of ≈200 fourth instar larvae [25]. The tissues were lyophilized and homogenized in 0.01 M potassium phosphate buffer (pH 7.4) with 0.45% (w/v) diethyldithiocarbamic acid (DIECA) and 0.2% (v/v) β-mercaptoethanol (2.5 ml of buffer per gram of larva), sonicated for 20 seconds and filtered through two layers of cheesecloth. The filtrate was loaded on to a 10, 30 and 60% discontinuous sucrose gradient and centrifuged 6 h at 60000 × *g*, 10°C. Fraction between 30 and 60% corresponding to the virus band, was collected and RNA was extracted using RNAzol^®^ RT (Sigma-Aldrich) following the manufacturer's protocol. Quantitative PCR (qPCR) was undertaken as described below to determine the prevalence of SeIV-1 and SeIV-2.

### Effect of iflaviruses on SeMNPV pathogenicity, virulence and adult persistence

To assess the effect of iflavirus co-infection on the insecticidal properties SeMNPV OBs, larvae were inoculated with mixtures of SeMNPV and SeIV-1 or SeIV-2. For this batches of pre-molt *S*. *exigua* second instars were starved overnight and, having molted, groups of 30 second instars were allowed to drink a suspension containing one of five OB concentrations using the droplet feeding method [35]. The OB concentrations were 0 (control), 2.54 × 10^5^, 8.18 × 10^4^, 2.72 × 10^4^, 9.09 × 10^3^ and 3.03 × 10^3^ OB/ml, that were previously demonstrated to kill between 95% and 5% of inoculated larvae. Identical groups of larvae were inoculated with OBs suspensions that contained (i) 10^9^ SeIV-1 genomes/μl, (ii) 10^9^ SeIV-2 genomes/μl, or (iii) 1:1 mixture of 5 × 10^8^ SeIV-1 and 5 × 10^8^ SeIV-2 genomes/μl. Larvae that consumed the suspension within 10 minutes were individually placed in the wells of a 24-compartment plates with a piece of semi-synthetic diet and reared at 25 ± 1°C. Virus-induced mortality was recorded at 8 h intervals for 7 days post-inoculation by which time all lethally infected larvae had died. The entire bioassay was performed three times.

OB concentration-mortality results were subjected to logit regression in GLIM 4 [36]. Relative potencies were estimated when a parallelism test confirmed that the regressions for each treatment could be fitted with a common slope. Time-mortality results for larvae inoculated with the highest OB concentration (2.54 × 10^5^ OB/ml) were subjected to Weibull survival analysis in GLIM 4. Larval that did not die from virus infections were not included in the analysis [37]. The validity of the Weibull model was determined by comparing fitted values with Kaplan–Meier survival function estimated values [38].

To determine the prevalence of SeMNPV infection in adults, the insects that survived in the treatment involving 2.72 × 10^4^ OB/ml were reared to the adult stage and examined by qPCR as describe bellow.

### Effects of iflaviruses on OB production and larval weight gain

To examine the effect of iflaviruses on OB production, larvae were treated with OB suspensions with or without the presence of SeIV-1 or SeIV-2. For this, groups of 24 newly-molted second instars were inoculated with 2.45 × 10^5^ OB/ml (previously estimated to result in 90% mortality) and one of the following iflavirus treatments: (i) 10^9^ SeIV-1 genomes/μl; (ii) 10^9^ SeIV-2 genomes/μl; and (iii) a 1:1 mixture of 5 × 10^8^ SeIV-1 and 5 × 10^8^ SeIV-2 genomes/μl. Two additional groups of 24 insects in the second instar were inoculated with SeMNPV OBs alone or were mock-infected as controls. Larvae that consumed inoculum suspension within 10 minutes were individually placed in the wells of 24 compartment plates and reared at 25 ± 1°C, checked daily for signs of disease. The entire bioassay was performed three times. Larval weight gain, and OB production were subsequently assessed in fourth instar larvae that were co-inoculated with 5 × 10^7^ OB/ml and 10^9^ SeIV-1 genomes/μl using the droplet-feeding method described above. This concentration of OBs was estimated to kill 95% of inoculated fourth instars. Weight measurements were taken using an electronic balance (±1 mg) immediately before inoculation and then at 24 h intervals during a period of 6 days. Larvae that had been inoculated in the second or fourth instar and that showed signs of the final stages of polyhedrosis disease, were placed individually in 1.5 ml microtubes and incubated at 25 ± 1°C for 4–8 hours until death. Virus-killed larvae were then stored at 4°C for up to 7 days prior to OB counting. Ten cadavers per treatment and repetition were randomly selected for OB counting. Virus-killed larvae were homogenized individually in 1 ml distilled water. OB production was estimated by counting triplicate samples of diluted OB suspension using a Neubauer hemocytometer (Hawksley, Lancing, UK) under a phase-contrast microscope at ×400 magnification. Mean OB production values were estimated and compared by t-test (SPSS v. 21.0 2012, IBM). Weight gain data were not normally distributed and therefore subjected to the Kruskal–Wallis or Mann-Whitney non-parametric test (SPSS v. 21.0 2012, IBM).

### Total DNA and RNA extraction

For detection of covert infections, total DNA and RNA were isolate from insect tissues using the Master Pure Complete DNA and RNA Purification kit (Epicentre Biotechnologies). The abdomens of frozen adults were dissected and placed individually in a 2 ml microfuge tube with ceramic beads, 300 μl tissue lysis solution and 1 μl proteinase K (50 ng/μl). Samples were homogenized using MP FastPrep-24 tissue cell homogenizer at 4 m/s for 20 s and incubated at 65°C for 15 min at 1000 rpm orbital agitation. Samples were divided in two aliquots of 150 μl each. One aliquot was used for DNA extraction and treated with 1 μl RNAse at 37°C for 30 min. Tissue remains were pelleted by adding protein precipitation reagent (MPC), vortexed, and centrifuged at 10000 × *g* for 10 min. DNA was precipitated from the supernatant with isopropanol, washed twice with 70% ethanol, and the pellet was resuspended in 20 μl milli-Q water and stored at -20°C. The second aliquot was used for RNA extraction. For this, protein precipitation reagent was added to the 150 μl aliquot, centrifuged at 10000 × *g* for 10 min and DNA was precipitated with isopropanol. Nucleic acid pellets were treated with RNAse-free DNAse buffer and 5 μl of DNAse for 30 min at 37°C. A 200 μl volume of 2 × T and C lysis solution was added and vortexed for 5 s followed by 200 μl of protein precipitation reagent and vortexed for 10 s. The debris was pelleted by centrifugation and the supernatant was washed once with isopropanol and twice with 70% ethanol. Finally, RNA was resuspended in 20 μl DEPC water and stored at -20°C. Blank extraction samples containing only water were processed in parallel to detect cross-contamination during the extraction process. All equipment and reagents were previously sterilized and treated with DEPC to remove RNases.

### Virus detection and quantification

In order to quantify viral loads, qPCR and reverse transcription quantitative PCR (RT-qPCR) were performed for SeMNPV and the iflaviruses, respectively. Specific primers were used for the detection of SeMNPV DNA polymerase [23] and iflavirus RNA-dependent RNA polymerase (RdRp) [24] sequences (Table 1). Total DNA or cDNA was used as template for amplification in order to detect SeMNPV and iflaviruses, respectively. To obtain cDNA, 1 μg of RNA was reversed transcribed to cDNA using SuperScript II Reverse Transcriptase (Promega) and Oligo (dT) primers. The reverse transcription mix consisted of 2 μl of 5× buffer (Promega), 1.2 μl MgCl_2_ (25 mM), 0.5 μl dNTP mix (10 mM), 0.8 μl DEPC water and 1 μl ImProm-II reverse transcriptase (Promega). The mixture was added to RNA samples and incubated at 25°C for 5 min, followed by 42°C for 60 min and 70°C for 15 min. qPCR based on SYBR Green fluorescence was carried out in a CFX96 Touch™ Real-Time PCR Detection System (Bio-Rad) in 96-well reaction plates. A 9 μl mastermix containing 5 μl SYBR, 0.5 μl of both primers (10 μM) and 3 μl water was added to 1 μl of DNA or cDNA template. For the construction of standard curves, the PCR products generated with specific primers for SeIV-1 and SeIV-2 were cloned into a pGEM®-T Easy cloning vector (Promega). Plasmid DNA was quantified using a spectrophotometer (Eppendorf BioPhotometer Plus) and two replicate samples were subjected to eight-fold serial dilutions in sterile MilliQ water (from 10^−1^ to 10^−7^ ng/μl) and were amplified as standards.

**Table 1:** Primer sequences and description of the gene region targeted.

Primer	Sequence	Description
qDNApol	F: 5’-CCGCTCGCCAACTACATTAC-3’ R: 5’-GAATCCGTGTCGCCGTATATC-3’	Amplifies a 149-bp fragment within the SeMNPV DNA polymerase gene [40].
SeIV-1q	F: 5’- TGTGAAGTTAGACACGCATGGAA-3’ R: 5’-CGACTTGTGCTACTCTCTTCATCAA-3’	Amplifies a 97-bp fragment in the RNA-dependent RNA polymerase (RdRp) region from SeIV-1 [12].
SeIV-2q	F: 5’-CCGCTCGCTTATTGAAACGT-3’ R: 5’-CATGAGACAGCTGGAATTGGAA-3’	Amplifies a 78-bp fragment in the RNA-dependent RNA polymerase (RdRp) region from SeIV-2 [13].
qATPsynthase	F: 5’-GTTGCTGGTCTGGTGGGATT-3’ R: 5’-AGGCCTCAGACACCATTGAAA-3’	Amplifies a 72-bp fragment in ATP-synthase subunit C gene from *S*. *exigua* [25].

The qPCR protocol consisted in an initial denaturation at 95°C for 3 min, followed by 45 amplification cycles of 95°C for 10 s, 62°C for 30 s, and finally a melting curve stage of 65°C to 95°C every 0.5°C for 5 s. Data were acquired and analyzed using Bio-Rad CFX Manager 3.1 software (Bio-Rad). The regression characteristics of the standard curves were R^2^ = 0.99 in all cases and slopes of -3.27, -3.56 and -3.30 for SeMNPV, SeIV-1 and SeIV-2, respectively, with an efficiency between 90 and 110% [39]. The last standard concentration, 10^−7^ ng/μl, represented the limit of detection and showed correct amplification curves and the expected melting temperatures of 83.5, 77.0 and 79.5°C for SeMNPV, SeIV-1 and SeIV-2, respectively. As a result, higher Ct values were considered to be virus-free samples. All values were normalized using a non-differentially expressed ATP-synthase subunit C gene quantification (Table 1), following standard procedures previously described for iflaviruses [25].

## Results

### Effect of iflavirus co-inoculation on SeMNPV insecticidal properties

In all cases, mortality increased significantly with log_e_ [OB concentration] (F_1,20_ = 108, *P*<0.001). Overall, mixtures of SeMNPV and iflaviruses resulted in significantly increased OB pathogenicity compared to SeMNPV OB inoculum alone. The LC_50_ values were 2.4 to 3.9-fold lower in iflavirus treatments compared to SeMNPV OBs alone (Table 2).

**Table 2:** LC_50_ values, relative potencies and mean time to death (MTD). For *S*. *exigua* second instars inoculated with SeMNPV OBs alone or co-infected with SeMNPV OBs and iflavirus (SeIV-1, SeIV-2 or SeIV-1+SeIV-2).

Treatment	LC_50_ (×10^3^ OBs/ml)	Relative potency	95% Fiducial limits		*P*	MTD (h)	95% Fiducial limits	
			Low	High			Low	High
SeMNPV alone	14.4	1	10.1	20.3	-	94.9ab	92.8	97.1
SeMNPV+SeIV-1	6.1	2.4	3.4	9.1	<0.05	94.4ab	92.1	96.8
SeMNPV+SeIV-2	3.7	3.9	1.3	6.5	<0.05	92.4a	90.4	94.5
SeMNPV+SeIV-1+SeIV-2	5.1	2.8	3.1	7.4	<0.05	97.1b	95	99.3

Mean time to death values of the different SeMNPV + iflavirus treatments ranged from 92.4 to 97.1 hours post inoculation (hpi), compared to 94.9 hpi for larvae inoculated with SeMNPV OBs alone (Table 2). MTD values only differed significantly between the SeMNPV + SeIV2 and the SeMNPV + SeIV-1 + SeIV-2 treatments (*t*-test = 3.679; df = 332; *P*<0.001). No mortality was registered in mock-infected larvae or in those larvae infected only with SeIV-1 or SeIV-2, or their mixture. All larvae that succumbed to SeMNPV infections showed the characteristic signs of polyhedrosis disease that was confirmed by microscopic observation of OBs.

### OB production and weight gain

**Fig 1:**
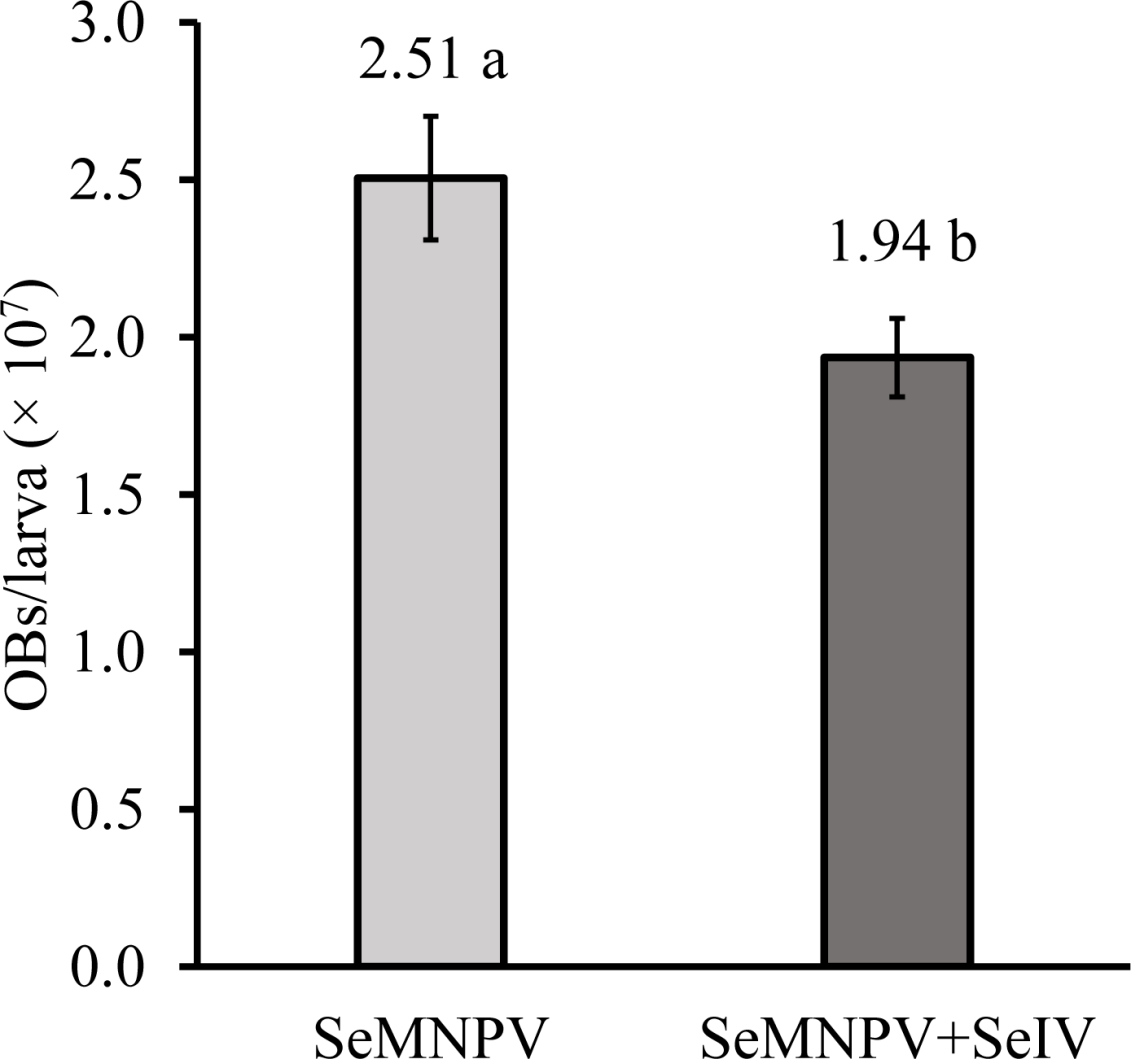
OB production in second instar larvae infected with SeMNPV alone or co-infected with iflaviruses. Iflavirus treatments did not differ significantly and were pooled for analysis. Mean values followed by different letters indicate significant differences (*t*-test = 2.335; df = 118; *P* = 0.01). Bars indicate the standard error.

Co-infection with iflavirus significantly reduced OB production in second instar larvae treated with SeMNPV (ANOVA, F_3,116_ = 4.008; *P*<0.001). The mean production of OBs in each SeMNPV-killed insect was similar in treatments involving SeIV-1 (1.89 × 10^7^ ± 1.96 × 10^6^), SeIV-2 (2.03 × 10^7^ ± 2.06 × 10^6^) or the mixture of SeIV-1 + SeIV-2 (1.89 × 10^7^ ± 2.10 × 10^6^) (ANOVA, F_2,87_ = 2.723; *P* = 0.07), so the results of these treatments were pooled for comparison of treatments with and without iflavirus. Overall, OB production per larva was reduced by 23% in larvae infected by SeMNPV + iflaviruses compared to larvae inoculated with SeMNPV OBs alone (*t*-test = 2.335; df = 118; *P* = 0.01) (Fig 1).

**Fig 2:**
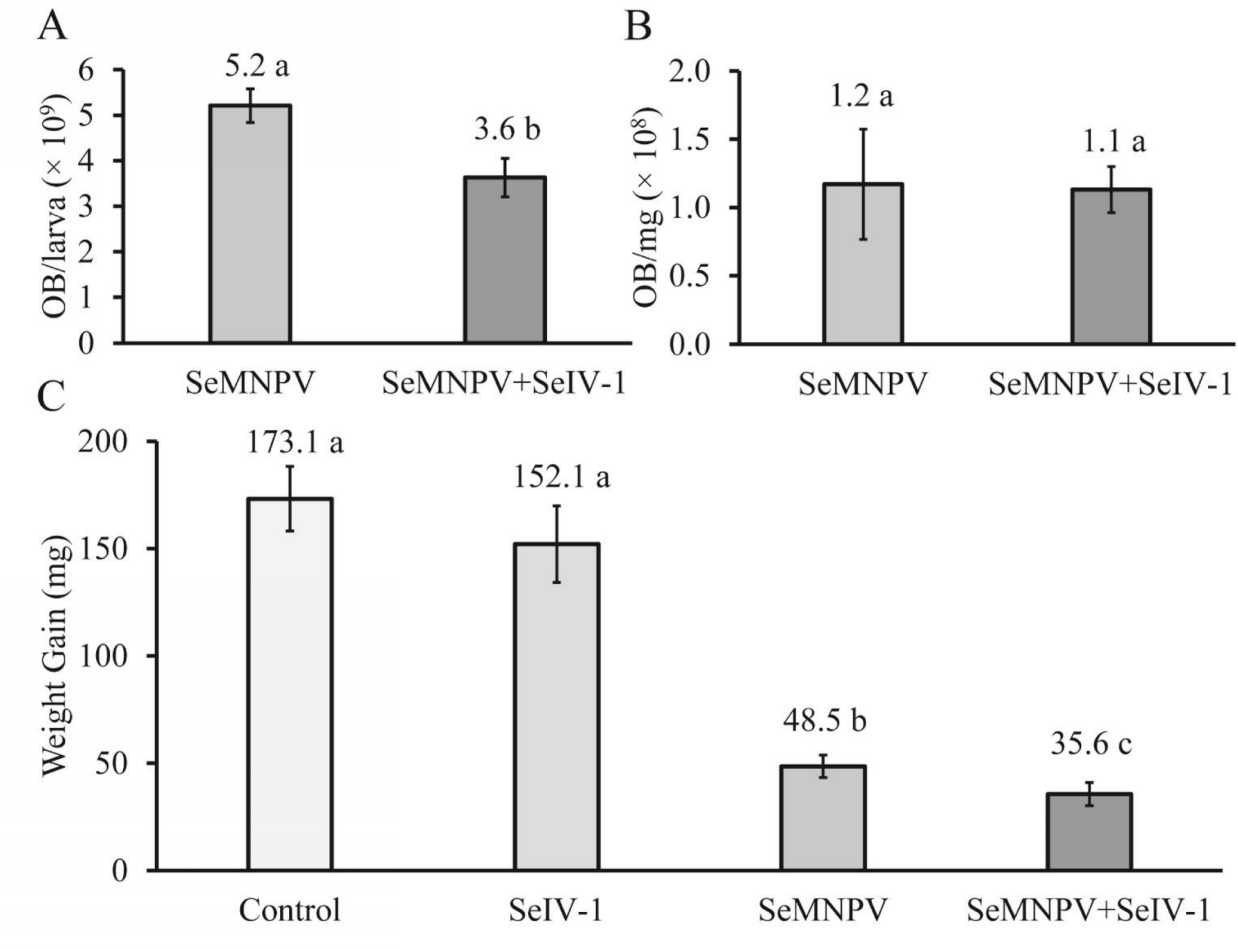
OB production and weight gain for fourth instar larvae treated with SeMNPV or SeMNPV + SeIV-1. Mean OB production (A), mean OB production per larval weight (mg) (B), and median weight gain of fourth instar larvae treated with water (control), SeIV-1, SeMNPV and SeMNPV + SeIV-1 (C). Columns headed by values with different letters indicate significant differences (Kruskal-Wallis; *P*<0.05). Bars indicate the standard error (A, B) or the interquartile range (C).

In order to determine whether the effects of iflavirus on OB production were related to larval growth, fourth instar larvae were subjected to one of two treatments: SeMNPV OBs alone and SeMNPV OBs + SeIV-1. Larval weights from 30 larvae per repetition and treatment was registered over time. For larval weight, two additional treatments, SeIV-1 alone and mock-infected larvae (control) were used. OB production in fourth instars differed significantly between treatments (F_1,133_ = 2.23; *P* < 0.001), with a 30% reduction in the production of OBs in insects infected by SeMNPV + SeIV-1 compared to larvae infected by SeMNPV alone (Fig 2A). However, OB production in relation to host weight (OB/mg body weight) was similar with or without SeIV-1 co-infection (F_1,129_ = 2.075; *P* = 0.152) (Fig 2B). These results can be explained by the significantly lower weight gain of larvae (27% reduction) when co-infected with SeMNPV and iflaviruses compared to larvae infected by SeMNPV alone (Kruskal-Wallis; χ^2^ = 82.03, df = 3; *P*<0.001) (Fig 2C).

### Detection and quantification of viral loads in sublethal SeMNPV and iflavirus infections

**Fig 3:**
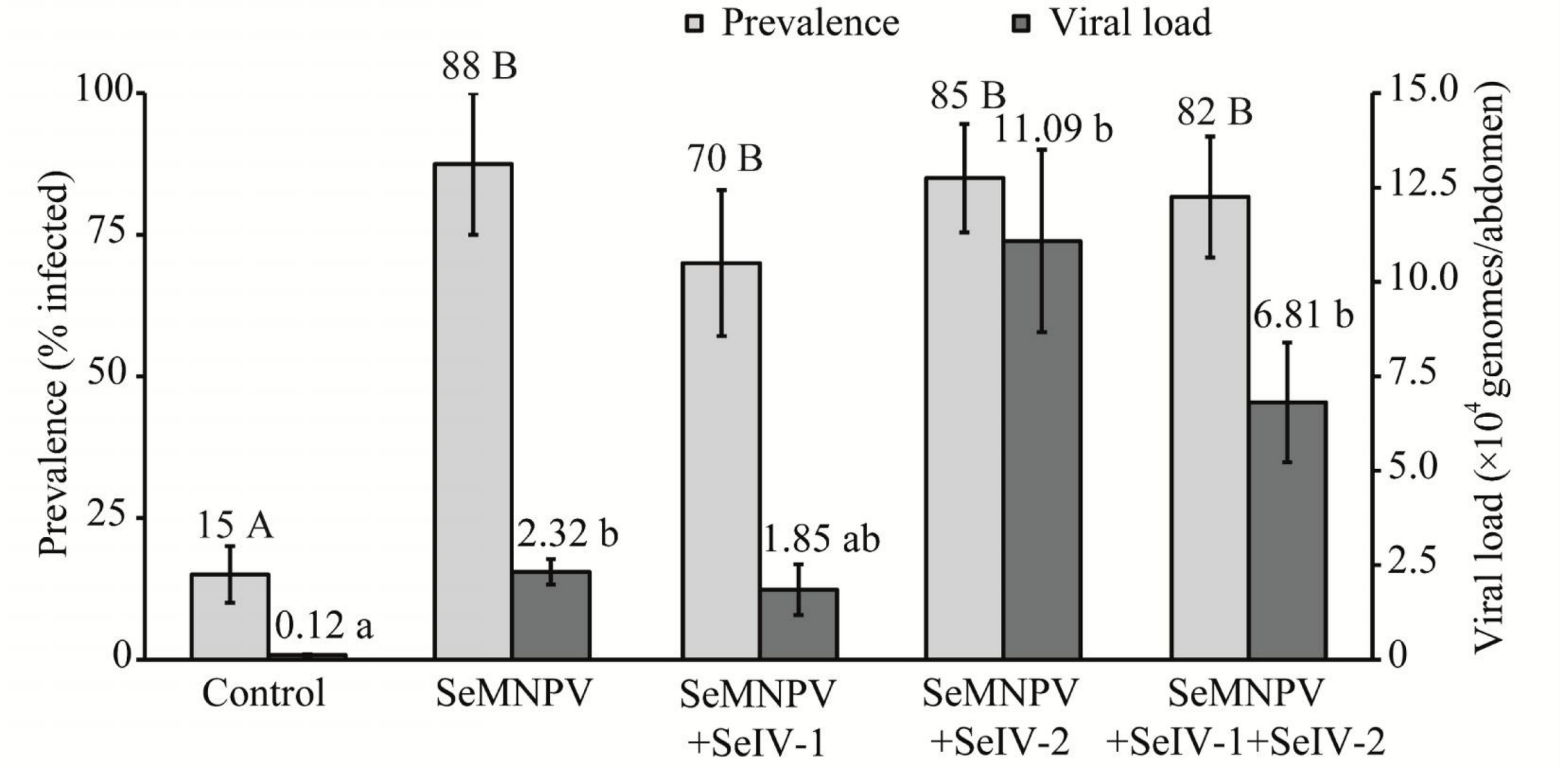
Prevalence of covert infection and viral load of SeMNPV in adults. Mean prevalence of covert infection by SeMNPV in adult survivors to a virus challenge (pale gray columns) and the viral load (dark shaded columns) of SeMNPV-positive adults by qPCR. Mean prevalence values followed by different upper case letters indicate significant differences between viral treatments (GLM; *P* < 0.05). Error bars indicate SE. Median values of viral load followed by different lower case letters differ significantly (Kruskal-Wallis; *P* < 0.05). Error bars indicate interquartile range (viral load).

Of the second instar larvae treated with a concentration of 9.09 × 10^3^ OBs/ml, 30 to 70% died of lethal polyhedrosis disease. The presence of sublethal infections was estimated by qPCR in adult survivors of this treatment. The prevalence of sublethal infection by SeMNPV was similar between larvae treated with SeMNPV with or without SeIV-1 or SeIV-2 and ranged from 70 to 88% (GLM; *P*<0.001) (Fig 3, pale shaded columns). Unexpectedly, low-levels of SeMNPV were detected in the control groups, probably due to the presence of a low-level persistent infection in the insect colony, as observed in a previous study [40]. Co-inoculation of SeMNPV+SeIV-2, or a mixture of SeIV-1 + SeIV-2, resulted in a significant increase in the SeMNPV load, which was over three-fold higher than SeMNPV loads registered in insects treated with SeMNPV + SeIV-1 or SeMNPV alone. A similar response was registered for the group inoculated with SeMNPV + SeIV-1 + SeIV-2 (Kruskal-Wallis; χ^2^ = 23.67; df = 4; *P*<0.001) (Fig 3, dark columns).

## Discussion

The effectiveness of baculovirus-based insecticides relies on a diversity of virus-related and environmental factors [14], but also on the susceptibility of the pest population to baculovirus applications, which can be altered by cryptic infections involving other types of viruses [23, 41]. Here we demonstrated that the insecticidal properties of SeMNPV OBs were altered when the baculovirus replicated in hosts that were also infected by the iflaviruses SeIV-1 or SeIV-2. Previous studies indicated that these iflaviruses could be present in 15% of field populations of *S*. *exigua* in southern Spain, in which 8.3% of individuals were simultaneously infected by both iflaviruses and SeMNPV [23].

As lethal disease has not been observed in insects infected by SeIV-1 or SeIV-2 to date, it appears that the co-inoculation of the iflaviruses increases the lethality of the SeMNPV infection. Similar effects on the mortality response have been observed following co-inoculation of mixtures of pathogens in lepidopteran larvae. For instance, the mixture of *Bacillus thuringiensis* (Bt) and Helicoverpa armigera nucleopolyhedrovirus (HaNPV) resulted in potentiation or additive effects on the mortality of *Plutella xylostella* that depended on the concentration of the bacteria in the mixture [42]. The gut microbiota of the host can also modulate baculovirus pathogenicity and speed of kill [43], as bacterial virulence factors seem to contribute to the success of viral infections in the insect midgut. Persistent infection by the endosymbiont *Wolbachia* also resulted in increased susceptibility to lethal nucleopolyhedrovirus infection in *Spodoptera exempta* [44]. These findings contrast with those involving persistent infection of *Helicoverpa armigera* larvae by a densovirus that provided resistance against both Bt and HaNPV infection [45]. Interestingly, densovirus infected individuals of *H*. *armigera* developed faster and had higher fecundity than healthy conspecifics [45], whereas in the present study, we observed lower weight gain in iflavirus infected larvae. This resulted in a reduced weight at death and a corresponding reduction in total OB production per larva compared to larvae infected with SeMNPV alone. The positive relationship between larval body weight and OB production is well established [46]. Our results also confirm previous findings on the reduced growth of iflavirus infected *S*. *exigua* larvae [24].

In a previous study on the interaction of SeMNPV OBs and SeIV-1, we observed that the inoculation of *S*. *exigua* larvae by SeIV-1-contaminated OBs resulted in a significant reduction in OB pathogenicity compared to OBs that were not contaminated by iflavirus, whereas OB production per larva and the weight gain of insects infected by SeMNPV were not significantly affected by the presence of SeIV-1 particles in the inocula [25]. It is difficult to compare these studies directly due to differences in the amounts of iflavirus present and the nature of the mixed virus inocula. Specifically, in our previous study insects were infected by both SeIV-1 and SeMNPV which resulted in the production of progeny SeIV-1 particles that were intimately associated with the polyhedrin matrix of OBs. The average (±SE) number of SeIV-1 particles per OB was estimated at 18.9±2.3 particles/OB [25]. In contrast, in the present study OBs and iflavirus particles were inoculated in mixtures in which the concentration of iflavirus remained fixed (10^9^ particles/μl) and the concentration of SeMNPV OBs varied. As such, the ratio of iflavirus particles to OBs varied from 4 × 10^6^ to 3.3 × 10^7^ SeIV particles per OB in the inocula consumed by second instar larvae, compared to 2 × 10^4^ SeIV-1 particles per OB in the inoculum fed to fourth instar larvae. As a result, the differences in larval weight gain and OB production are likely to be due to the higher concentrations of iflavirus particles present in inocula mixtures with OBs used in the present study compared with our previous study [25].

Similarly, the apparent contradiction between the present study that detected increased SeMNPV OB pathogenicity in mixtures with iflavirus, and our previous study in which SeMNPV OB pathogenicity was reduced when in association with iflavirus, may have been due to the higher quantity of iflavirus particles per OB in the inocula used to infect insects in the present study, or related to the physical association of iflavirus with OBs in the previous study [25]. Alternatively, OBs may have undergone modifications during assembly in cells co-infected by iflaviruses, which affected the physical features or number or infectivity of occlusion derived virions occluded within each OB [25]. These issues can only be resolved through future studies.

The concentration of iflavirus used in the present study may initially appear unrealistically high. However, recent studies on an iflavirus of *Helicoverpa armigera* revealed the presence of up to 10^6^ iflavirus genomes/μg of larval feces [47]. This was sufficient to assure the horizontal transmission of the iflavirus to conspecific larvae. Moreover, as quantitative studies have suggested that nucleopolyhedrovirus OBs are usually present at low densities on plant foliage in natural and agricultural ecosystems [48], small numbers of OBs may be consumed together with large quantities of iflavirus particles released from iflavirus-infected insects feeding on the same plant, although this has not been demonstrated empirically in field or laboratory studies.

In studies performed prior to the advent of molecular techniques, ELISA was used to detect a picorna-like virus that severely stunted the growth of *Trichoplusia ni* larvae, although no virus-specific mortality was observed [19, 20]. Mixed infections of this virus with Autographa californica multiple nucleopolyhedrovirus (AcMNPV) resulted in contamination of progeny OBs with the picorna-like virus [20]. The molecular mechanisms by which iflaviruses and baculoviruses interact are unclear, more so because SeMNPV replicates in the cell nucleus, whereas iflaviruses replicate in the cytoplasm [11].

Recent advances in recognizing the regulatory role of host and viral microRNAs (miRNA) in virus pathogenesis and virulence are likely to greatly improve our understanding of the molecular mechanisms that modulate insect host-virus interactions [49]. Virus-encoded miRNAs have been characterized in nucleopolyhedrovirus [50, 51] and RNA virus infections [52], in addition to a suite of miRNA-mediated antiviral and apoptotic responses by host cells [53–55]. The mechanisms by which SeMNPV or iflaviruses may be able to modulate the innate immune responses of the host through miRNA-mediated suppression strategies are presently unclear although both sublethal infection and interspecific virus interference are likely to be regulated to some degree through such mechanisms [54, 56].

SeMNPV infections are transmitted both horizontally from infected to healthy larvae and vertically from parents to offspring [57]. The persistence of the virus in sublethally infected adults may also contribute to transgenerational pest suppression if a portion of the sublethal infections are reactivated to cause lethal disease [58, 59]. In contrast, the transmission of iflaviruses is believed to be mainly horizontal [11]. However, we previously observed that SeIV-1 and SeIV-2 were efficiently transmitted from field-caught infected adults to their offspring in the laboratory [23]. Both the prevalence of sublethal infection by SeMNPV and viral load (number of copies of SeMNPV genomes) were influenced by the presence of iflaviruses in adult insects. Notably, the abundance of SeMNPV genomes in insects from treatments involving SeIV-2 (SeMNPV + SeIV-2 and SeMNPV + SeIV-1 + SeIV-2) were several fold higher than SeMNPV loads in insects from treatments involving SeMNPV alone or SeMNPV + SeIV-1. High loads of nucleopolyhedrovirus may promote transmission either vertically to offspring [40] or horizontally through the activation of sublethal infections [59]. In either case, once established, the intimate association between iflavirus and SeMNPV would likely be maintained, either through physical association of iflavirus particles and OBs or by dual infection of host offspring through simultaneous vertical transmission by both viruses [25].

Cryptic infections such as those caused by iflaviruses could modulate the performance of SeMNPV-based insecticides. As both types of viruses occur in natural populations of *S*. *exigua* nucleopolyhedrovirus-iflavirus interactions are likely to be common in nature. Iflaviruses are not desirable in insect colonies used for large scale production, as they may result in diminished larval growth, reduced OB production and, depending on their host-range, potential risks to non-target Lepidoptera present in treated crops and habitats adjacent to treated areas. As such, strict colony hygiene, frequent disinfection measures, regular quality control assays on OB pathogenicity and molecular screening for the presence of iflavirus contaminants are likely to be necessary in baculovirus mass-production facilities to maintain production of an effective product with registration-compliant levels of contaminant microorganisms.

## Supporting information

S1 File Original data from all experiments. (XLSX)
